# Benzo[a]pyrene disrupts LH/hCG-dependent mouse Leydig cell steroidogenesis through receptor/Gαs protein targeting

**DOI:** 10.1038/s41598-024-51516-7

**Published:** 2024-01-08

**Authors:** Clara Lazzaretti, Neena Roy, Elia Paradiso, Chiara Capponi, Tommaso Ferrari, Francesca Reggianini, Samantha Sperduti, Carmela Perri, Lara Baschieri, Elisa Mascolo, Manuela Varani, Giulia Canu, Tommaso Trenti, Alessia Nicoli, Daria Morini, Francesca Iannotti, Maria Teresa Villani, Elena Vicini, Manuela Simoni, Livio Casarini

**Affiliations:** 1 Unit of Endocrinology, Department of Biomedical, Metabolic and Neural Sciences, Baggiovara Hospital, University of Modena and Reggio Emilia, via Pietro Giardini 1355, 41126 Modena, Italy; 2 Department of Obstetrics, Gynecology, and Reproductive Sciences, University of California, San Diego, La Jolla, CA 92093 USA; 3 Center for Genomic Research, University of Modena and Reggio Emilia, Via Giuseppe Campi 287, 41125 Modena, Italy; 4 International PhD School in Clinical and Experimental Medicine (CEM), University of Modena and Reggio Emilia, Via Giuseppe Campi 287, 41125 Modena, Italy; 5 Department of Laboratory Medicine and Pathology, Azienda USL/Azienda Ospedaliero-Universitaria di Modena, 41126 Modena, Italy; 6 Department of Obstetrics and Gynaecology, Fertility Center, ASMN, Azienda Unità Sanitaria Locale-IRCCS di Reggio Emilia, 42123 Reggio Emilia, Italy; 7 Department of Anatomy, Histology, Forensic Medicine and Orthopedic, Section of Histology, La Sapienza University, Rome, Italy; 8 Unit of Endocrinology, Department of Medical Specialties, Azienda Ospedaliero-Universitaria di Modena, Modena, Italy

**Keywords:** Endocrine reproductive disorders, Gonadal hormones

## Abstract

Steroidogenesis of gonadal cells is tightly regulated by gonadotropins. However, certain polycyclic aromatic hydrocarbons, including Benzo[a]pyrene (BaP), induce reproductive toxicity. Several existing studies have considered higher than environmentally relevant concentrations of BaP on male and female steroidogenesis following long-term exposure. Also, the impact of short-term exposure to BaP on gonadotropin-stimulated cells is understudied. Therefore, we evaluated the effect of 1 nM and 1 µM BaP on luteinizing hormone/choriogonadotropin (LH/hCG)-mediated signalling in two steroidogenic cell models, i.e. the mouse tumor Leydig cell line mLTC1, and the human primary granulosa lutein cells (hGLC) post 8- and 24-h exposure. Cell signalling studies were performed by homogeneous time-resolved fluorescence (HTRF) assay, bioluminescence energy transfer (BRET) and Western blotting, while immunostainings and immunoassays were used for intracellular protein expression and steroidogenesis analyses, respectively. BaP decreased cAMP production in gonadotropin-stimulated mLTC1 interfering with Gαs activation. Therefore, decrease in gonadotropin-mediated CREB phosphorylation in mLTC1 treated with 1 μM BaP was observed, while StAR protein levels in gonadotropin-stimulated mLTC1 cells were unaffected by BaP. Further, BaP decreased LH- and hCG-mediated progesterone production in mLTC1. Contrastingly, BaP failed to mediate any change in cAMP, genes and proteins of steroidogenic machinery and steroidogenesis of gonadotropin-treated hGLC. Our results indicate that short-term exposure to BaP significantly impairs steroidogenic signalling in mLTC1 interfering with Gαs. These findings could have a significant impact on our understanding of the mechanism of reproductive toxicity by endocrine disruptors.

## Introduction

Human reproductive functions are regulated by gonadotropins, which mediate the proliferation, differentiation, and steroidogenesis of steroidogenic cells. The binding of luteinizing hormone (LH) or human chorionic gonadotropin (hCG) to luteinizing hormone/choriogonadotropin receptor (LHCGR) results in the activation of Gαs protein which, in turn, activates cyclic adenosine monophosphate (cAMP)/protein kinase A (PKA) pathway, leading to phosphorylation of extracellular-regulated kinase 1/2 (pERK1/2) and cAMP response-element binding protein (CREB), mediating steroidogenesis and gonadal cell proliferation^1,2^.

Benzo[a]pyrene (BaP) is a polycyclic aromatic hydrocarbon generated through the incomplete combustion of fossil fuels, wood, and other organic materials and is also found in diesel exhaust, cigarette smoke, barbecued/grilled/broiled and smoke-cured foods, and industrial waste by-products^3^. It is known to induce reproductive toxicity in both men and women^4–8^. For instance, analysis of semen samples from 86 Sicilian volunteers for BaP markers, i.e. BaP Tetrol I-1 (TI-1) and BaP Tetrol II-2 (TII-2), found an inverse correlation between sperm motility and both the compounds, suggesting that BaP exposure may negatively affect male fertility^9^. Animal studies have also shown the endocrine-disrupting properties of BaP in reproductive functions. Sub-acute and sub-chronic exposure of male rats with BaP via inhalation resulted in reduced testosterone and impaired epididymal function^10,11^. Moreover, long-term oral exposure to BaP in rats resulted in decreased epididymal sperm quality and low testosterone levels^12^. Female rats exposed to 50, 75, or 100 µg BaP/m^3^ via inhalation 4 h a day, for 14 days, showed reduced plasma levels of estradiol and LH at the proestrus stage and reduced progesterone at diestrus I. Also, the number of pups per litter as well as ovulation rate decreased after exposure to 100 µg BaP/m^3^ indicating reduced ovarian function^13^. Isolated rat follicles treated with BaP, at doses representative of those measured in human follicular fluid^14^, inhibited the follicle growth and reduced estradiol production^15^. Taken together, these data suggest that long-term exposure to BaP may interfere with steroidogenesis, negatively impacting reproductive functions.

Despite the wide literature on BaP impact on fertility, its short-term effects on gonadal cells are still poorly investigated. In this in vitro study, we evaluated if short-term (< 24 h) exposure to BaP interferes with gonadotropin-mediated intracellular signalling pathways and steroidogenesis, in two cell models: the mouse tumor Leydig cell line mLTC1 and the human primary granulosa lutein cells (hGLC). mLTC1 is one of the best Leydig cell model available so far, given the paucity of human male gonadal cells. mLTC1 cells have mouse LH receptors (Lhr), as well as steroidogenic response upon treatment with human LH and hCG^16^. However, differences between mLTC1 and human steroidogenesis exist, such as relatively marked progesterone production detected in the mouse cell line^17^. hGLC is a validated in vitro model to study the human ovarian steroidogenesis^18–20^. This study helps us to dissect the molecular mechanisms of the short-term effects of BaP in gonadotropin-stimulated cells.

## Results

### Cell viability assay for BaP dose-finding

A cell viability assay using 3-(4,5-dimethylthiazol-2-yl)-2,5-diphenyltetrazolium bromide (MTT) was performed to select the BaP concentrations to be used in vitro. Both mLTC1 and hGLC were treated with BaP concentrations ranging between 1 fM and 1 mM, and we found that 1 mM BaP was toxic to cells (Supplementary Fig. 1). Therefore, we decided to choose the non-toxic 1 nM and 1 µM BaP concentrations, which were reported to be relevant to dietary^21–24^ and smoking exposures^14^.

### BaP decreases cAMP production in mLTC1 but not in hGLC

**Figure 1:**
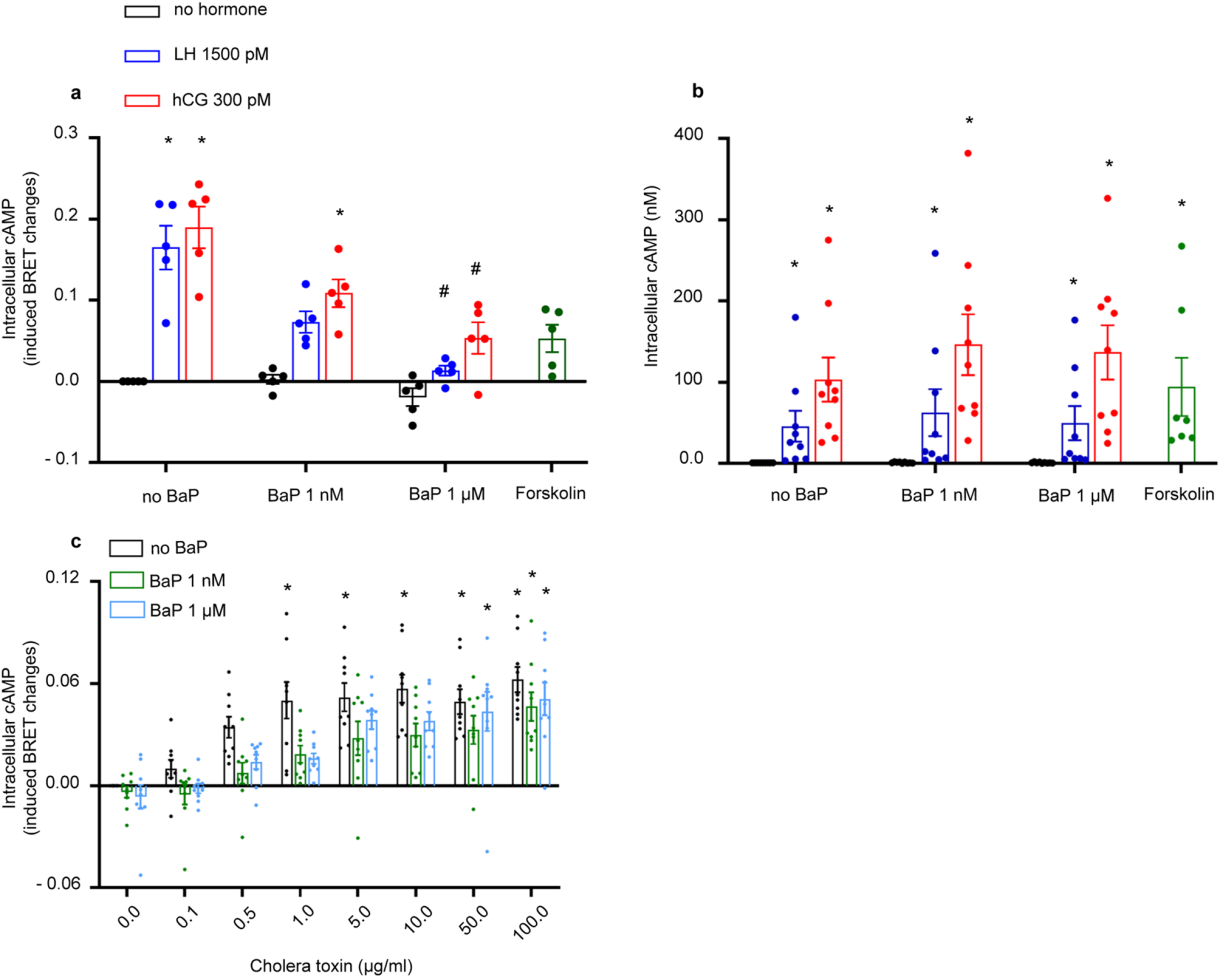
BaP effect on cAMP response in mLTC1 and hGLC. Effect of BaP on LH- and hCG-induced cAMP response in mouse Leydig cells (mLTC1) analysed by BRET assay (**a**) (n = 5) and in human granulosa cells (hGLC) measured by analysed HTRF assay (**b**) (n = 9). Forskolin was used as a positive control. (**c**) Analysis of cAMP production under cholera toxin treatment in the presence of BaP by BRET in mLTC1 (n = 9). Data are represented as mean ± SEM. CTX = cholera toxin. *, Significantly different *vs* “no hormone”; # “no BaP” *vs* “BaP” (Kruskal Wallis test and Dunn’s post-hoc test, *p < 0.05).

We assessed the effect of BaP on LH- and hCG-induced cAMP production, in mLTC1 and hGLC. Cells were exposed to 1500 pM LH and 300 pM hCG, as concentrations which were previously described to induce the maximal intracellular cAMP accumulation^18,25,26^, in the presence or in the absence of 1 nM and 1 µM BaP for 30 min. 50 µM forskolin was used as a positive control in hGLC and mLTC1 cells, before intracellular cAMP measurement. We observed a decrease in cAMP production in gonadotropin-stimulated mLTC1 co-treated with BaP 1 nM (Fig. 1a; Kruskal Wallis test, *p* ≥ 0.05). Further, BaP at 1 µM significantly decreased cAMP production in gonadotropin-stimulated mLTC1 compared to gonadotropin treatments alone (Fig. 1a; Kruskal Wallis test and Dunn’s post-hoc test, *p* < 0.05). Conversely, in hGLC, we did not observe any change in cAMP levels induced by LH or hCG alone or in co-treatment with 1 nM and 1 µM of BaP (Fig. 1b; Kruskal Wallis test, *p* ≥ 0.05).

To identify the mechanism through which BaP inhibits the cAMP response in mLTC1, we investigated cAMP production under cholera toxin (CTX)/BaP and forskolin/BaP co-treatment. CTX and forskolin are direct activators of Gαs and adenylate cyclase, respectively. Two BaP concentrations (1 nM and 1 µM) were tested. While 0.1–100 µg/ml CTX induced dose-dependent intracellular cAMP accumulation, the presence of BaP lead to significant decrease in cAMP production, compared to CTX alone (Fig. 1c; Kruskal Wallis test and Dunn’s post-hoc test, *p* < 0.05). Also, mLTC1 was co-treated with 50 µM forskolin and BaP. However, we did not find any significant effect of BaP on cAMP production, in forskolin-stimulated mLTC1 (Supplementary Fig. 2b; Kruskal Wallis test and Dunn’s post-hoc test, *p* < 0.05). These results suggest that, in mLTC1, BaP interferes with Gαs protein functioning, but not with adenylate cyclase, decreasing cAMP production.

In hGLC, the absence of BaP effects on LH/hCG- induced cAMP was further confirmed by treating cells with forskolin together with 1 nM and 1 µM BaP. The presence of the endocrine disruptor did not impact forskolin-induced cAMP production (Supplementary Fig. 2c; Kruskal Wallis test, *p* ≥ 0.05). Interestingly, even cholera toxin-induced cAMP response  was not affected by BaP co-treatment (Supplementary Fig. 2a; Kruskal Wallis test, *p* ≥ 0.05), suggesting that short-term exposure to this compound does not impair cAMP/PKA pathway activation in granulosa cells.

### BaP disrupts CREB phosphorylation in mLTC1 cells

**Figure 2:**
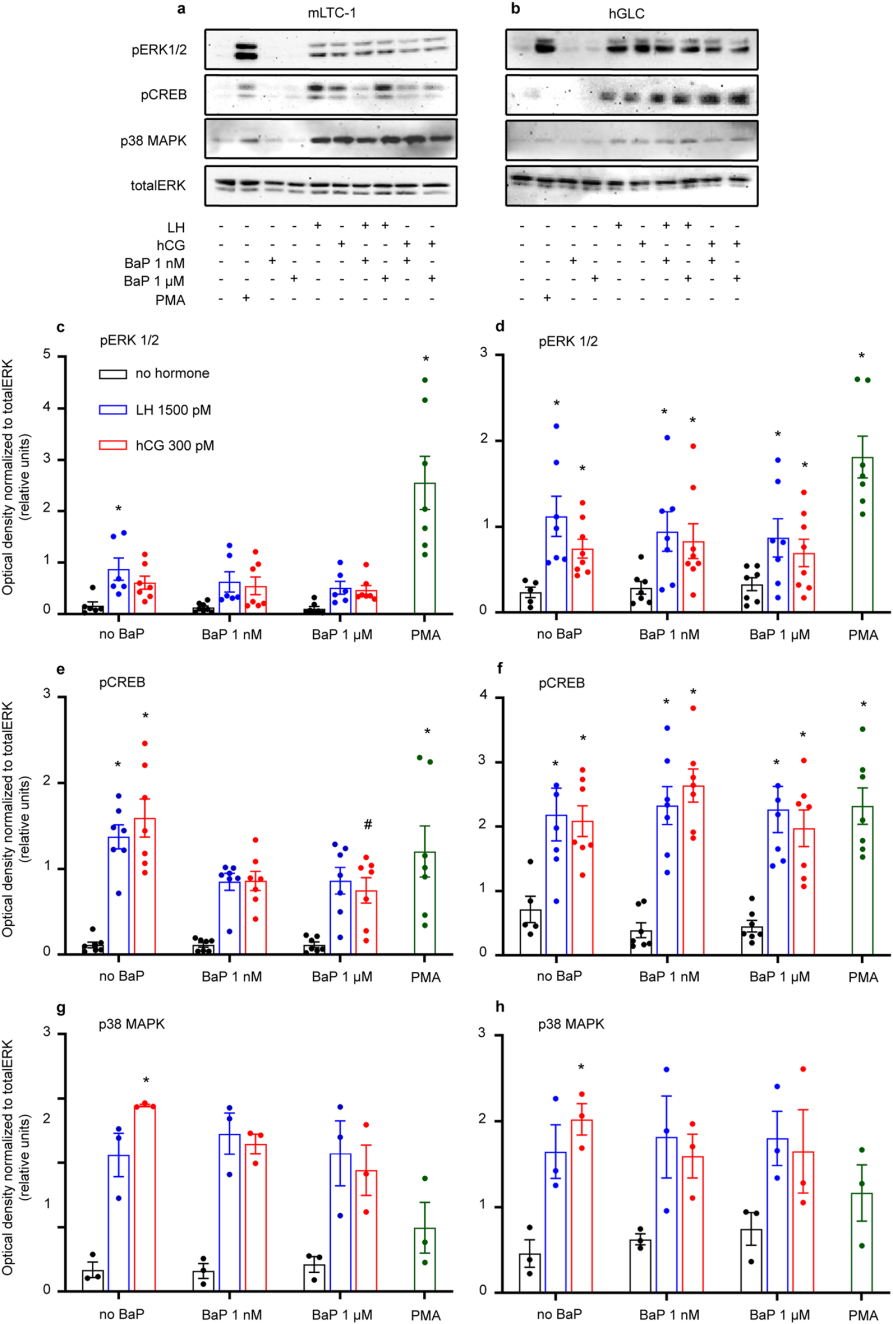
Effect of BaP on ERK 1/2, CREB and 38MAPK phosphorylation in mLTC1 and hGLC cells. Representative images of western blotting analyses performed in mLTC1 (**a**) and in hGLC (**b**) after BaP exposure in the presence or in the absence of hCG or LH. Relative semi-quantification of pERK 1/2 (**c**,**d**) (n = 7), pCREB (**e**,**f**) (n = 7) and p38MAPK (**g**,**h**) (n = 3) activation in mLTC1 and hGLC cells respectively. Data are represented as mean ± SEM. *, Significantly different *vs* “no hormone”; # “no BaP” *vs* “BaP” (Kruskal Wallis test and Dunn’s post-hoc test, *p < 0.05). In western blot images contrast was adjusted where required. Uncropped original western blotting images are presented in Supplementary Fig. 7.

In mLTC1 and hGLC, we assessed the effect of BaP on cAMP-dependent intracellular events triggered by LH/hCG, i.e. ERK1/2, CREB, and p38MAPK phosphorylation. In both cell lines, treatment with gonadotropins induced the phosphorylation of ERK1/2, CREB, and p38MAPK compared to controls (Fig. 2; Kruskal Wallis test and Dunn’s post-hoc test, *p* < 0.05). In mLTC1, the administration of 1 nM and 1 μM BaP did not change the LH- and hCG-induced ERK1/2 and p38MAPK phosphorylation (Fig. 2a,c and g; Kruskal Wallis test, *p* ≥ 0.05). However, a decrease in gonadotropin-mediated CREB phosphorylation was found, in hCG-treated mLTC1 exposed to 1 μM BaP (Fig. 2e; Kruskal Wallis test and Dunn’s post-hoc test, *p* < 0.05). Instead, no changes in ERK1/2, CREB, and p38MAPK phosphorylation were detected, in gonadotropin-stimulated hGLC co-treated with 1 nM and 1 μM BaP, compared to gonadotropins alone (Fig. 2b,d,f,h; Kruskal Wallis test, *p* ≥ 0.05).

### StAR protein expression level is unaffected by BaP

**Figure 3:**
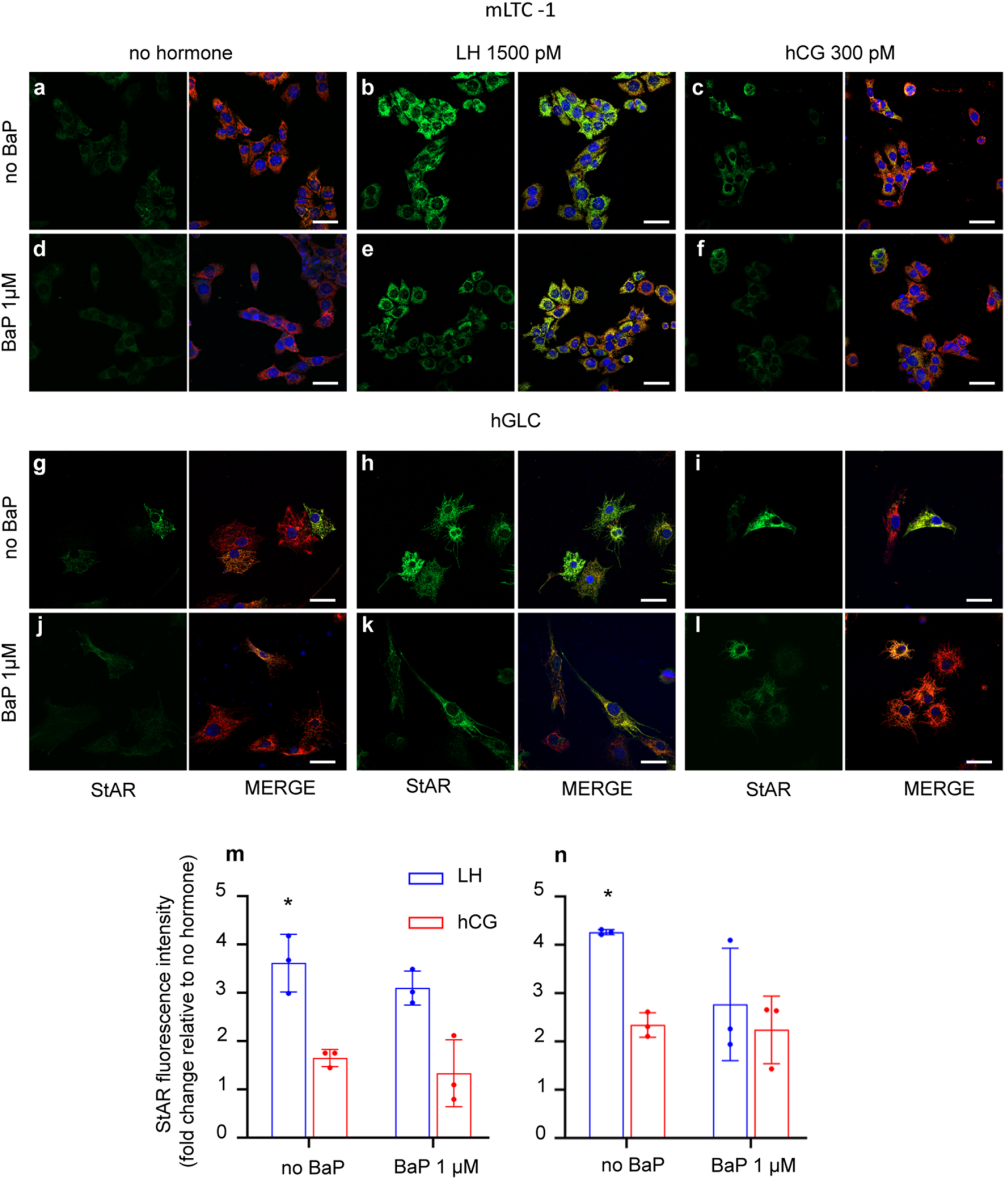
BaP effect on StAR expression. Representative immunofluorescence images showing the effect of 1 uM BaP on StAR protein expression (in green) in mLTC1 cells (**a**–**f**) and hGLC cells (**g**–**l**) after 24 h of gonadotropins treatment. MERGE shows StAR protein (green), the mitochondrial marker HSP60 (red), and nuclei (blue, TOPRO3). Relative quantification of StAR fluorescence intensity in mLTC1 (**m**) and hGLC (**n**). n = 3. Scale bar: 50 μm. * Significantly different vs “no hormone” (Kruskal Wallis test and Dunn’s post-hoc test, *p < 0.05.

Given the inhibitory activity of BaP on cAMP/PKA pathway in mLTC1, we evaluated the effect of this endocrine disruptor on steroidogenic acute regulatory protein (StAR) expression, which is directly involved in steroid production^27,28^. To this end, mLTC1 and hGLC were co-treated 24 h with gonadotropins with or without BaP, and StAR protein levels were evaluated by immunostaining (Fig. 3). In these experiments, StAR subcellular localization was evaluated by co-staining with the mitochondria heat shock protein 60 (Hsp60), a mitochondrial marker. Our results demonstrated that StAR co-localized to mitochondria in both cell types (Supplementary Fig. 3). We found that cell treatment with LH increased StAR protein levels in both cell lines (Fig. 3b,h,m and n; Kruskal Wallis test and Dunn’s post-hoc test, *p* < 0.05), while hCG induced a protein fold change increase of 1.7 and 2.3 times in mLTC1 and hGLC, respectively, even though no statistical difference was found (Fig. 3c,i,m and n; Kruskal Wallis test, p < 0.05). However, the co-treatment with BaP did not affect StAR immunoreactivity, in both cell types (Fig. 3d–f and j–l). Western blot analysis on StAR protein expression level in mLTC1 confirmed the data obtained by immunostaining (Supplementary Fig. 4). At the transcriptional level, we confirmed that in mLTC1 cells, LH increased StAR-coding gene (*Stard1*) expression^27,28^ and *Cyp17a1* compared to unstimulated cells at both 8 and 24 h of stimulation (Supplementary Fig. 5a and c; Kruskal Wallis test and Dunn’s post-hoc test, *p* < 0.05), while hCG increased *Stard1* expression only at 8 but not 24 h (p < 0.05). Similarly, in hGLC, LH and hCG treatment induced the expression of *STARD1* and *CYP19A1*, but not of *CYP17A1*, both at 8 h and 24 h (Supplementary Fig. 6; Kruskal Wallis test, *p* < 0.05). Interestingly, in line with the immunostaining results, co-treatment of mLTC1 and hGLC with gonadotropins and BaP did not impact *Stard1* gene expression, as well as the other steroidogenesis-related genes analyzed (p ≥ 0.05).

### BaP decreases progesterone production in mLTC1 cells

**Figure 4:**
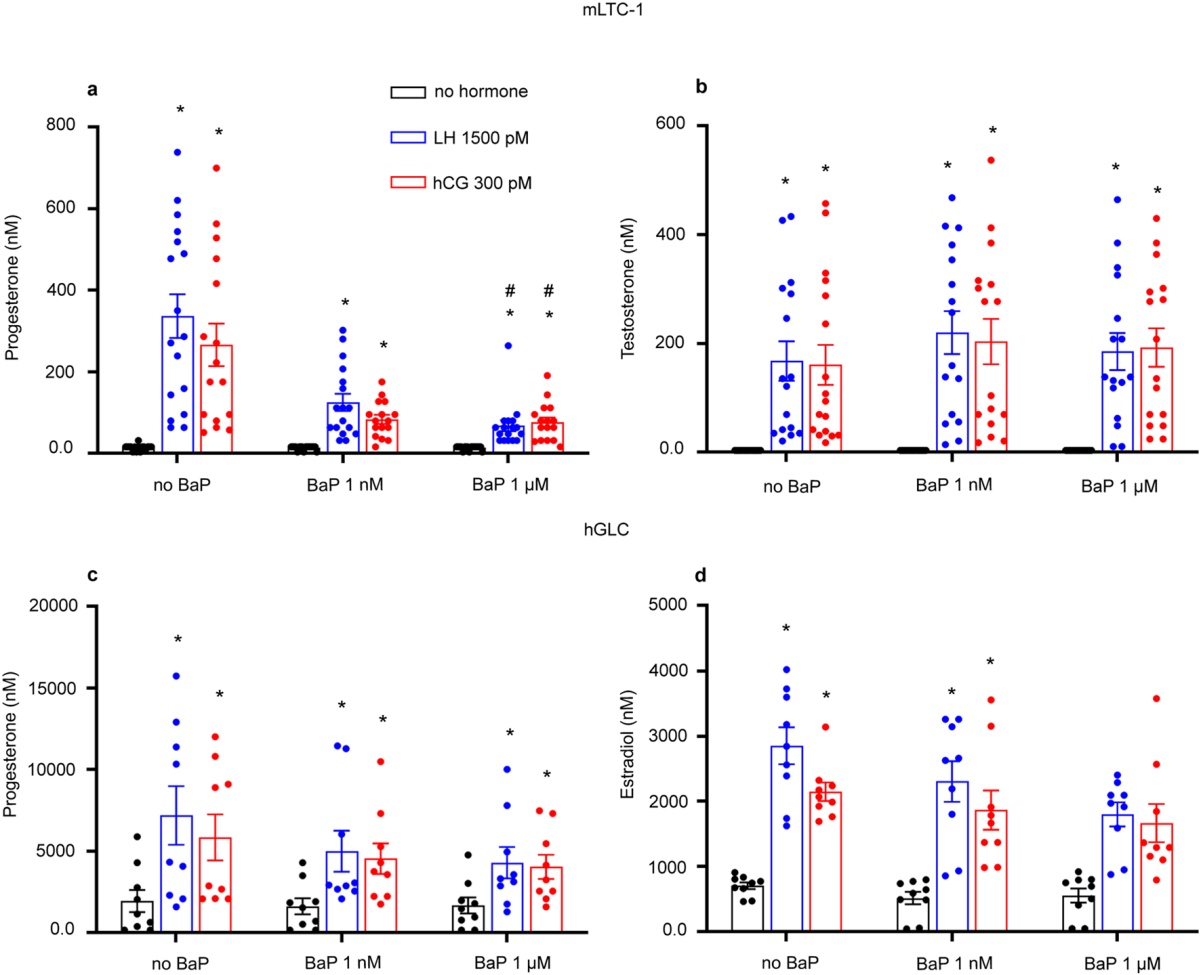
BaP effect on 24 h-steroids production in mLTC1 and hGLC. Effect of BaP on gonadotropins-induced progesterone (**a**) and testosterone (**b**) levels in mLTC1 (n = 16) and progesterone (**c**) and estradiol (**d**) levels in hGLC (n = 9) after 24 h treatment. Data are represented as mean ± SEM. *, Significantly different vs. “no hormone”; # “no BaP” *vs* “BaP” (Kruskal Wallis test and Dunn’s post-hoc test, *p < 0.05).

We investigated the effect of BaP on 8- and 24-h LH/hCG-induced steroidogenesis, by measuring the progesterone and testosterone levels in mLTC1, and progesterone and estradiol levels in hGLC. In mLTC1 cells, LH and hCG significantly increased progesterone levels compared to untreated cells at both 8 and 24 h of stimulation (Fig. 4a and supplementary Fig. 6a; Kruskal Wallis test and Dunn’s post-hoc test, *p* < 0.05). Interestingly, 1 μM BaP significantly inhibited gonadotropin-induced progesterone production at 24 h (Fig. 4a) but not at 8 h (Supplementary Fig. 7a; Kruskal Wallis test and Dunn’s post-hoc test, *p* < 0.05). Similarly, testosterone levels significantly increased following 8- and 24-h treatment with gonadotropins compared to unstimulated cells (Fig. 4b and Supplementary Fig. 7b; Kruskal Wallis test and Dunn’s post-hoc test, *p* < 0.05), while BaP failed to induce any effect at both time points (Fig. 4b and Supplementary Fig. 7b; Kruskal Wallis test, *p* ≥ 0.05).

In hGLC, we detected an increase in both progesterone and estradiol levels after 8-h treatment with gonadotropins (Supplementary Fig. 7c and d; Kruskal Wallis test, *p* ≥ 0.05), as well as after 24 h (Fig. 4c and d; Kruskal Wallis test and Dunn’s post-hoc test, *p* < 0.05). However, BaP failed to mediate any change to both steroid levels in the presence of gonadotropins (Fig. 4c and d and Supplementary Fig. 7c and d; Kruskal Wallis test, *p* ≥ 0.05).

## Discussion

**Figure 5:**
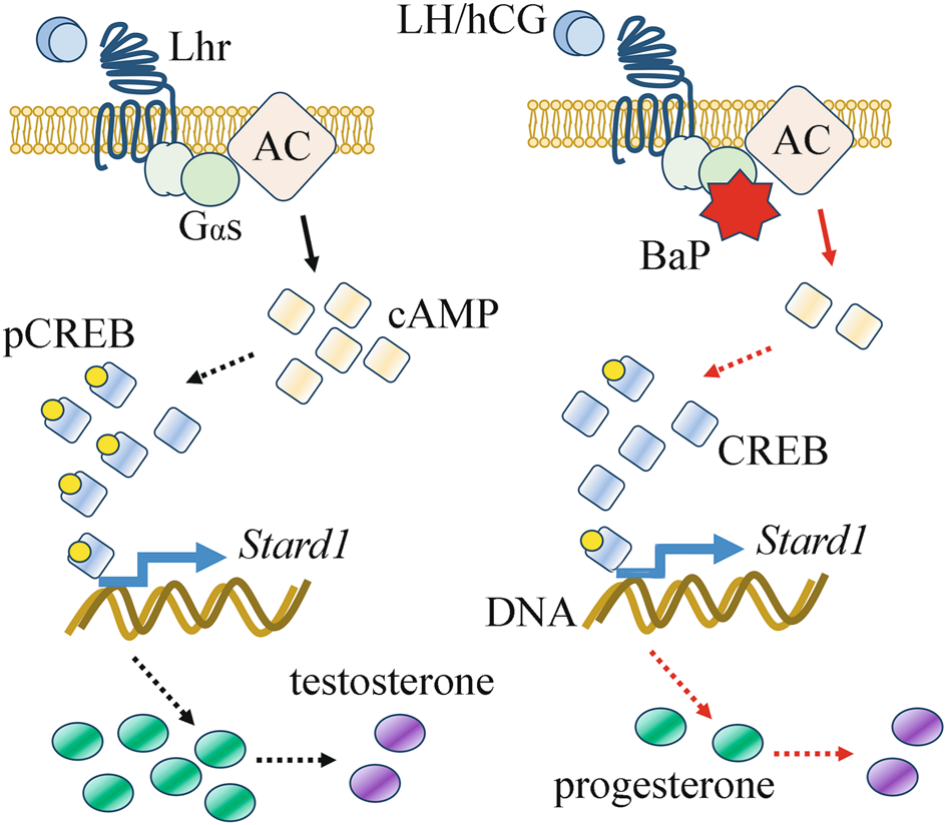
Schematic representation of BaP action in mLTC1 cells. LH and hCG bind Lhr expresed in the cell surface, which modulates a cascade of Gαs protein-mediated intracellular events, i.e. adenylyl cyclase (AC) activation, cAMP increase, CREB phosphorylation, upregulation of Stard1 gene expression, progesterone and testosterone synthesis. BaP interaction with Gαs protein decreases cAMP production and related downstream events, leading to lower progesterone levels.

In the present study, we investigated the effect of BaP on gonadotropin-induced steroidogenic signalling, in models of male and female gonadal steroidogenic cells, i.e. mLTC1 and hGLCs. For this purpose, we co-exposed cells to BaP and LH/hCG, and evaluated cAMP production and related intracellular signalling pathways, gene and protein expression, and steroidogenesis. We demonstrated that BaP significantly affects cAMP production and progesterone synthesis in mLTC1 by impairing Gαs protein activation, downstream steroidogenic intracellular signalling and progesterone synthesis, whereas hGLC remains unaffected at the BaP doses used. Our results indicate that short-term exposure to BaP significantly impairs steroidogenic signalling in mLTC1 (Fig. 5).

BaP detected in women ranges from 0.40 ± 0.13 ng/mL (1.6 ± 0.5 nM), in serum, and 1.32 ± 0.68 ng/mL (5.2 ± 2.7 nM) in follicular fluid of smoking women undergoing in vitro fertilization treatment^15^. In men, the urinary concentration of BaP ranged from 0.05 ng/L (0.2 pM), in the control group, to 12.5 ng/L (49.5 pM) in those exposed to smoke due to occupation^29^. In the present study, we have used human exposure-relevant BaP concentrations to see their effect in gonadal cells. We observed that cAMP production decreased in mLTC1 when they were co-treated with LH/hCG and BaP (Fig. 1). Further, we assessed the target of BaP, linked to cAMP decrease, by treating mLTC1 with cholera toxin and forskolin. While no significant decrease in cAMP levels were found when cells were co-treated with forskolin and BaP, exposure to BaP and cholera toxin decreased cAMP levels compared to cholera toxin alone. These results, together with the short-term nature of the BaP effect, suggest that the endocrine disruptor decreases cAMP production in mLTC1 directly targeting the LH receptor or Gαs protein, rather than decreasing the receptor expression levels. We may hypothesize that the direct action of BaP on GPCR/Gαs protein could be responsible of disrupted early signalling also of other receptors^30–32^. A similar mechanism of action was previously described for BaP-induced interference with the β2-adrenoreceptor (β2ADR)/Gαs protein/cAMP signalling cascade^33^. In that case, BaP was demonstrated to bind β2ADR at a polycyclic aromatic hydrocarbon site of the molecule, likely inducing a conformational change upregulating Gαs protein and cAMP production^33^. In summary, our results are consistent with a rapid interfering effect operated by BaP at the receptor/Gαs protein module, impacting the related signalling pathway. However, we can not exclude that other molecules are involved in the BaP action, such as the aryl hydrocarbon receptor (AhR)^34^, which was identified as a receptor of the endocrine disruptor. In vivo, AhR mediates even carcinogenic events of BaP, effectively inhibited by receptor knock-out. However, these effects would occur after relatively long treatments, which could not be necessarily consistent with rapid G protein signalling.

The phosphorylation of signalling molecules downstream to cAMP, such as ERK1/2, CREB, and p38MAPK, was evaluated (Fig. 2). Consistently with cAMP data, we observed a decrease in CREB phosphorylation in mLTC1, but not in hGLC. The different activity of BaP on the two cell types might be explained by different sequences between mouse and human LH receptors^25^, which could lead to different binding affinity for BaP. Also, cell-specific expression levels of receptors, Gαs proteins, and molecules modulating GPCR recycling could impact the action of BaP^35^. The presence of LHCGR putative membrane partners in hGLC, such as the follicle-stimulating hormone receptor or the G protein-coupled estrogen receptor^36,37^, could allosterically counterbalance BaP inhibitory action and recover the cAMP pathway activation. Finally, we could hypothesize that BaP might have intracellular targets, such as the protein kinase C or phosphodiesterases^38,39^, whose activity potentially influences the crosstalk among GPCR signalling network partners. Therefore, there are multiple candidates that may explain the cell-specific BaP mode of action and that need investigation.

The cAMP/PKA signalling pathway upregulates the expression of steroidogenesis-related genes and proteins^2^ (Fig. 3 and Supplementary Figs. 5 and 6). Thus, we assessed the expression of *STARD1,* which codes the StAR protein involved in the transport of cholesterol from the cytoplasm to the inner mitochondrial membrane^40^. In mLTC1, *Stard1* expression did not cange as instead was demonstrated for cAMP and pCREB activation. This inconsistency could be due to multiple, cAMP-dependent intracellular regulators acting as an on/off switch for *Stard1* expression. We further measured the effect of BaP on steroidogenic events depending on StAR, i.e. progesterone and testosterone synthesis^28^. While no differences in progesterone levels after 8 h of co-treatment with BaP and gonadotropins were detected, in mLTC1, progesterone levels decreased in the presence of the endocrine disruptor after 24 h of co-treatment. Interestingly, testosterone levels did not change after BaP and gonadotropin co-treatment. Decrease of progesterone levels should not be due to the ability of BaP to perturb StAR protein expression levels (Fig. 3), which did not change according to mRNA levels. This inconsistency could be due to mismatch between mRNA and protein levels^41,42^. Rather, in mLTC1, we may hypothesize that BaP could interfere with StAR activity, reducing the cholesterol intake into the mitochondria and subsequently decreasing progesterone production (Fig. 4). Unfortunately, although some steps forward in the comprehension of StAR functioning were done^43–45^, its molecular mechanism of action is largely unknown^46^ and further analyses are needed to verify our hypothesis. As an alternative, the presence of BaP could lead to upregulation of the *Cyp17a1* gene. *Cyp17a1* encodes for Steroid 17-alpha-hydroxylase/17,20 lyase involved in the conversion of progesterone to testosterone^47^. A similar effect was repored previously^48^, when mLTC1 treatment with p,p′DDT lead to reduction of progesterone production. However, in that case there was no change in testosterone secretion and authors suggested that this may be due to up-regulation of *CYP17* gene as reported earlier^49^. The reason for the no change in testosterone may be due to the cell model that we used. In literature, it is established that progesterone is the main steroid produced by mLTC1, and testosterone is produced in very low amounts as the original tumor cell M5480 produces progesterone and very low testosterone^16,50^. Therefore, mLTC1 could be a good model to study the effect of BaP on the induction of steroidogenesis. Contrastingly, in hGLC, BaP did not influence the 8- and 24-h expression of StAR protein and steroidogenesis-related genes, occurring likely as a function of results indicating no effects on Gαs protein activation. The lack of BaP inhibition on progesterone and estradiol production is consistent with cell signalling, StAR protein and gene expression data.

The origin of differences between hGLC and mLTC1 susceptibility to BaP is unclear and could be matter of further studies. We may hypothesize that different receptor/Gαs protein expression levels in the two cell models could impact the efficacy of BaP in disrupting gonadotropin signalling. Moreover, BaP action could be modulated by other membrane molecules not characterized herein, differently expressed between hGLC and mLTC1, and impacting the endocrine disruptor efficacy. However, it is also plausible that defective signals due to BaP action on LH receptor may be rescued by intermolecular cooperation with other GPCRs^51^, such as the follicle-stimulating hormone receptor (FSHR), which is expressed in hGLC but not in Leydig cells^2^. In this case, cAMP signalling could be activated through FSHR via interaction with BaP-targeted LHCGR, resulting in normal hGLC steroidogenesis, while it would be not in mLTC1. Finally, the aminoacid sequence diversity between human and mouse LH receptors^25^ could possibly be at the origin of different binding affinity to BaP, resulting in cell-specific effects.

In conclusion, BaP is a chemical compound potentially affecting Leydig cell functions and steroidogenesis even under short-term exposure at environmental concentrations. The effect is cell-specific since the compound failed in inducing the same disruption in ovarian gonadal cells. These data point out the sex-specific susceptibility to endocrine disruptors.

## Material and methods

### Patients’ selection

Human primary granulosa lutein cells (hGLC) were collected from the follicular fluid aspirate of women undergoing oocyte retrieval for assisted reproduction at the Santa Maria Nuova hospital (Reggio Emilia, Italy). Patients matched the following criteria: no endocrine abnormalities; no severe viral or bacterial infections; aged between 25 and 45 years. All the experiments and methods performed were in accordance with the relevant guidelines and regulations. Patients gave their written informed consent for the study. The study was approved by the local Ethics Committee (documents ‘Protocollo n. 2017/0015890 del 26/06/2017’, ‘Protocollo n2018/0080377 del 16/07/2018’, ‘Protocollo n. 2018/0080389 del 16/07/2018’, ‘Protocollo n. 2019/0009846 del 24/01/2019’ and ‘Protocollo n. 2019/0048906 del 23/04/2019’) and conformed to the principles outlined in the Declaration of Helsinki.

### Isolation and culture of human granulosa lutein cells

hGLCs were isolated from follicular fluids as previously described^18,19,52^. Briefly, hGLCs were purified using a 50% Percoll gradient (GE Healthcare, Little Chalfont, UK) and centrifuged. Red blood cell contamination was removed by adding hemolysis buffer and then medium containing DMEM/F12 (Gibco, Thermo Fisher Scientific, Waltham, MA, USA), 10% fetal bovine serum (FBS), 2 mM L-glutamine, 100 IU/mL penicillin, 0.1 mg/mL streptomycin (all from Thermo Fisher Scientific, Waltham, MA, USA) and 250 ng/ml Fungizone (Merck KGaA, Darmstadt, Germany) was added to stop this reaction. Following centrifugation, hGLCs were washed in Dulbecco’s phosphate-buffered saline (DPBS; Merck KGaA). Finally, hGLCs were resuspended and cultured in complete media for 6 days to allow them to recover gonadotropin receptor expression^52^. The cells were then serum-starved overnight before any analyses.

### mLTC1 cell line

mLTC-1 cells (ATCC CRL-2065, LCG Standards, Molsheim, France) were grown in RPMI 1640 medium (Gibco, Thermo Fisher Scientific) supplemented with 10% FBS, 100 IU/mL penicillin, 0.1 mg/mL streptomycin, 2 mM glutamine, and 1 mM HEPES at 37 °C and 5% CO_2_. mLTC1 cells were seeded and subsequently serum-starved for 12 h before any treatments.

### Cell viability assay

mLTC-1 and hGLC were seeded in a density of 1.5 × 10^4^ and cultured in a 96-well plate. After an overnight starvation, cells were treated with 1 fM, 1 pM, 1 nM, 1 µM and 1 mM of Benzo[a]pyrene (BaP) (Sigma-Aldrich, St. Louis, Missouri, US) and incubated for 12, 24 and 48 h at 37 °C and 5% CO_2_. Thereafter, cells were washed in DPBS and incubated with 3-(4,5-dimethylthiazol-2-yl)-2,5-diphenyltetrazolium bromide (MTT) (1 mg/mL) solution (Sigma-Aldrich). After 3 h of incubation, MTT solution was removed, and isopropanol (100 µL/well) was added to solubilize the formazan crystals. The absorbance was measured at 565 nm.

### Analysis of intracellular cAMP production by homogeneous time resolved fluorescence (HTRF)

hGLC and mLTC1 were seeded and cultured in 96-well plates (2 × 10^4^ cells/well). Prior to the stimulation, the cells were treated with the phosphodiesterase inhibitor 3-isobutyl-1-methylxanthine (IBMX) (Sigma-Aldrich) for 20 min. hGLC cells were then exposed 3 h to 1 nM and 1 µM BaP, in the presence or absence of the 3xEC_50_ of LH (1500 pM) and hCG (300 pM)^18,25,26^. A 50 µM dose of Forskolin (Sigma-Aldrich) was used as a positive control. To analyze the impact of BaP on adenylate cyclase activation, mLTC1 and hGLC were stimulated with 50 µM Forskolin in the presence or in the absence of 1 nM and 1 µM BaP, for 1 and 3 h, respectively. For the analysis of BaP effect on Gαs activation in hGLC, cells were treated 3 h with increasing doses of cholera toxin (CTX) (Sigma-Aldrich) (0.1–100 µg/ml) in the presence or in the absence of 1 nM and 1 µM BaP. Cell media were removed after treatments cells were rapidly frozen at − 80 °C to stop the reaction. This was followed by collecting the cells in 30 µl of the phosphate buffered saline (PBS) and the total cAMP produced was measured by cAMP-Gs Dynamic kit following the manufacturer’s instruction (Cisbio, Codolet, France).

### Analysis of intracellular cAMP accumulation by bioluminescence resonance energy transfer (BRET) in mLTC1

For cAMP analysis, 2 × 10^4^ mLTC1 were seeded in a 96-well plate and transiently transfected with 100 ng/well of the previously described cAMP biosensor Camyel-expressing vector^53,54^ mixed with 0.5 μl/well of Metafectene PRO (Biontex Laboratories GmbH, München, Germany) in serum-free medium. After 48 h, the medium was removed and 40 μl/well of DPBS added of 1 mM HEPES (ThermoFisher Scientific) and 500 µM of IBMX were administrated to the cells for 20 min. Cells were then exposed to LH (1500 pM) and hCG (300 pM), 50 µM Forskolin or CTX in the range of 0.1–100 µg/ml, in the presence or in the absence of 1 nM and 1 µM BaP for 30 min. Please, note that forskolin could not serve as a proper positive control for cAMP production in the mLTC1 cell line, due to low efficacy in this model^55^. Eventually, 5 µM coelenterazine H (Interchim, Montluçon, France) was added. Emissions from donor and acceptor were detected at wavelengths of 480 ± 20 and 540 ± 20 nm, respectively using the CLARIOstar microplate reader (BMG Labtech, Ortenberg, Germany).

### Western blotting

hGLC cells and mLTC1 cells were seeded and cultured in a 24-well plate (1 × 10^5^ cells/well) and then serum starved for 12 h before the stimulation. The cells were treated with 1 nM and 1 µM BaP in the presence or absence of 3xEC_50_ LH and hCG, and the positive control phorbol 12-myristate 13-acetate (PMA) (Sigma-Aldrich) for 15 min. The analysis of StAR expression levels in mLTC1 was performed after exposing the cells to 1 µM BaP in the presence or in the absence of hCG or LH for 24 h. Untreated cells were used as negative controls. Media containing the stimuli was removed and cells were immediately lysed in RIPA Laemmli buffer containing protease and phosphatase inhibitors. Validated specific antibodies and protocols for western blotting were used to evaluate phospho-p38 MAPK, phospho-ERK1/2, phospho-CREB. Total ERK1/2 was used as a normalizer, since it has similar expression levels than other target proteins, as previously validated^18,25,56^. StAR was evaluated by incubating the membranes with a specific antibody (1:500; sc-166821, Santa Cruz Biotechnology, Santa Cruz, CA, USA), while β-actin was used as a normalizer (1:40 000, A3854, Sigma‐Aldrich). A chemiluminescent detection solution (Cyanagen, Bologna, Italy) was used for signal development and the image was acquired by an image analysis system (VersaDoc Imaging System and QuantityOne software, Bio-Rad Laboratories, Inc., Hercules, CA, USA). Signals were then semi-quantified using the ImageJ software (National Institutes of Health, USA).

### Gene expression

5 × 10^4^ cells/well of hGLC and mLTC1 cells were seeded and cultured in a 24-well plate and serum-starved overnight. Cells were treated with BaP at 1 nM and 1 µM concentrations, in the presence or in the absence of 3xEC_50_ LH and hCG for 8 and 24 h. Media containing stimuli was then removed and the cells were immediately frozen at − 80 °C. Total RNA was extracted by phenol–chloroform method using RNA Extracol (EURx Sp. z o.o., Gdańsk, Poland). The total RNA was then reverse transcribed using Multiscribe reverse transcriptase (Applied Biosystems, Thermo Fisher Scientific, Waltham, MA, USA), and the gene expression was evaluated by real-time PCR. The following genes were evaluated in hGLC: *STARD1* (fw 5′-AAGAGGGCTGGAAGAAGGAG-3′; rev 5′-TCTCCTTGACATTGGGGTTC-3′), *CYP17A1* (fw 5′-AGCCGCACACCAACTATCAG-3′; rev 5′-GCAAACTCACCGATGCTGGA-3′) and *CYP19A1* (fw 5′-TACATTATAACATCACCAGCATCG-3′; rev 5′-TCATAATTCCACACCAAGAGAA-3′). The genes evaluated in mLTC1 were *Stard1* (fw 5′-ACAGACTCTATGAAGAACTT-3′; rev 5′-GACCTTGATCTCCTTGAC-3′) and *Cyp17a1* (fw 5′-CGAACACCGTCTTTCAATGACC-3′; rev 5′-TGGCAAACTCTCCAATGCTG-3′). The real-time data was normalized to the endogenous control: *RPS7* (fw 5′-AATCTTTGTTCCCGTTCCTCA-3′; rev 5′-CGAGTTGGCTTAGGCAGAA-3′) for hGLC^36^, and *Hprt1* (fw 5′-GCGTCGTGATTAGCGATGATG-3′; rev 5′-TCTCGAGCAAGTCTTTCAGTCC-3′) for mLTC1^25^.

### Immunofluorescence

3.5 × 10^4^ cells/well were seeded onto 3-chamber slides and serum-starved 12 h before treatment. The cells were treated with 1 µM BaP, in the presence or absence of 3xEC_50_ LH/hCG. The treatment was done in the presence of 1 µM androstenedione for hGLCs, during all the conditions. The media was removed after 24 h, and cells were washed in PBS, fixed in 4% paraformaldehyde (PFA) (Electron Microscopy Sciences) for 10 min at 4 °C, washed twice with PBS, and incubated with 0.5% TritonX‐100 (Sigma‐Aldrich) for 10 min at room temperature (RT). The reduction of nonspecific background signal was obtained by incubating cells with 1 M glycine (Sigma‐Aldrich) and subsequently with 5% normal donkey serum (Jackson Laboratories Immuno Research) block solution for 30 min at RT. Cells were incubated with mouse anti‐StAR antibody (1:100, sc-166821, Santa Cruz Biotechnology) for 2 h at RT and anti-HSP60 antibody (1:100, sc-1052, Santa Cruz Biotechnology), then with the secondary antibody Alexa Fluor® 488 AffiniPure Donkey Anti‐Mouse IgG (H + L) (1:200, #715‐545‐150, Jackson Immuno Research) and Cy™3 AffiniPure Donkey Anti-Goat IgG (H + L) (1:200, #705-165-147, Jackson Immuno Research) for 1 h at RT. Following extensive washes in PBS, cells were counterstained with DAPI (Invitrogen) and mounted in Vectashield mounting medium (Vector Laboratories). Pictures were acquired using a Zeiss Airyscan 2 confocal microscope. The mean fluorescence intensity (MFI) of individual cells was quantified on stored pictures using LAS AF Software or ZEN 3.2 as previously described^57^. To evaluate the MFI, we first selected and measured three different non-fluorescent regions of interest (ROI) from the same image to obtain the mean value of the background’s MFI. This background value was then subtracted from the MFI of ROIs manually draw around single immunoreactive cells. For each condition, at least 100 cells were randomly selected and analyzed from three different experiments.

### Analysis of steroidogenesis

hGLC and mLTC1 were seeded in 48-well plates at a concentration of 3 × 10^4^ cells/well and serum-starved for 12 h before treatment. The cells were treated with 1 nM and 1 µM BaP in the presence or absence of 3xEC_50_ LH and hCG, 8 and 24 h. hGLC stimulation media were added of 1 µM androstenedione (Sigma-Aldrich), under all the conditions. The plates containing the media were rapidly frozen at − 80 °C. Later, progesterone and estradiol concentrations were analysed in hGLCs, and progesterone and testosterone were analysed in mLTC1. The analysis was carried out performing a chemiluminescence immunoassays (CLIA) using the DXI800 instrument (Beckman Coulter, CA, USA). More in detail, progesterone was measured using the Access Progesterone kit (Beckman Coulter) (sensitivity of 0.1 ng/ml), estradiol with the Access Sensitive Estradiol kit (Beckman Coulter) (Limit of Quantification- LOQ 19 pg/ml) and testosterone with Access Testosterone kit (Beckman Coulter) (sensitivity of 0.1 ng/ml).

### Statistical analysis

Statistics were performed using GraphPad Prism 5 (GraPhPad Software Inc., San Diego, CA, USA). Results were analysed applying the Kruskal–Wallis test and the Dunn’s post-hoc test, after testing for normality by D’Agostino and Pearson test. Values of *p* < 0.05 were considered significant.

## Conclusions

In this study, we demonstrated that short-term exposure to BaP, administered at human exposure-relevant concentrations, decrease the progesterone response to LH/hCG via a molecular mechanism involving Gs protein activation. While these effects were detected in mLTC1, BaP did not affect the hGLC response to gonadotropins. This study emphasizes the need to deepen investigations on molecular mechanisms explaining possible, short-term and sex-specific deleterious effects of endocrine disruptors on reproductive health.

### Supplementary Information

Supplementary Legends.

Supplementary Figure 1.

Supplementary Figure 2.

Supplementary Figure 3.

Supplementary Figure 4.

Supplementary Figure 5.

Supplementary Figure 6.

Supplementary Figure 7.

Supplementary Information.

## Data Availability

All data generated or analysed during this study are included in this published article (and its Supplementary Information files).
