# Supplementary material for: Benzo[a]pyrene disrupts LH/hCG-dependent mouse Leydig cell steroidogenesis through receptor/Gαs protein targeting
Source: Sci Rep. 2024 Jan 8;14:844. doi: 10.1038/s41598-024-51516-7 (PMC10774265; doi:10.1038/s41598-024-51516-7)
Supplement: Supplementary file 1 — Supplementary Legends. [file 41598_2024_51516_MOESM1_ESM.docx]

**Supplementary figure captions**

**Supplementary Figure 1. BaP effect on mLTC1 and in hGLC cell viability.** Cell viability was measured by MTT assay in mLTC1 (a) and in hGLC (b) treated with increasing doses (1 fM – 1 mM) of BaP for 12, 24 and 48 h. Data are represented as mean ± SEM. (Kruskal Wallis test; p > 0.05; n=3).

**Supplementary Figure 2. Characterization of BaP activity on Gαs and adenilate cyclase functiong.** (a) The intracellular cAMP production was analysed in hGLC cells treated with increasing concentrations of cholera toxin in the presence or in the absence of 1 nM or 1 µM BaP (n=3). The Forskolin-induced cAMP response was measured in mltc1 (b) and hGLC (c) by HTRF assay in the presence of BaP (n=5). Data were represented as mean ± SEM. *, Significantly different vs “no Forskolin”; (Kruskal Wallis test and Dunn’s post-hoc test, *p < 0.05).

**Supplementary Figure 3. StAR localize to the mitocondrial compartment.** Representative immunofluorescence images of StAR (green), HSP60 (red) and nuclei (TOPRO3) in mLTC1 cells (a) and hGLC cells (b) after 24 h of gonadotropins treatment. Scale bar: 25µm.

**Supplementary Figure 4. Effect of BaP on StAR enzyme expression levels, in mLTC1.** Representative images of Western blotting analyses for StAR expression levels. Cells were exposed 24 to 1 µM BaP, and hCG or LH. Membranes were incubated with a specific antibody anti-StAR, producing a 30 KDa band (a), while β-actin was used as a normalizer (b). Samples were loaded in the following sequence: 1 = no hormone and no BaP, 2 = LH 1500 pM, 3= hCG 300 pM, 4 = BaP 1 µM, 5 = LH 1500 pM + BaP 1 µM, 6 = hCG 300 pM + BaP 1 µM. Western blot analysis confirmed results obtained by immunostaining as indicated by relative semi-quantification of StAR expression over β-actin (1 = 0.67 ± 0.20; 2 = 1.61 ± 0.33; 3 = 1.42 ± 0.29; 4 = 0.47 ± 0.15; 5 = 0.81 ± 0.10; 6 = 2.83 ± 1.50; Kruskal Wallis test and Dunn’s post-hoc test, p > 0.05; n=3).

**Supplementary Figure 5. Effect of BaP on the expression of genes involved in steroidogenic activity in mLTC1 cells.** Stard1 (a, c) and Cyp17a1 (b, d) expression were analysed in mLTC1 after 8 (a,b) and 24 h (c,d) of gonadotropins treatment in the presence or in the absence of BaP. Data are represented as ratio over the housekeeping gene Hprt1 (mean ± SEM ). *, Significantly different vs “no hormone”; # “no BaP” vs “BaP” (Kruskal Wallis test and Dunn’s post-hoc test, *p < 0.05; n=6).

**Supplementary Figure 6. Gene expression analysis under gonadotropin and BaP treatment in hGLC.** The expression of STARD1 (a,b), CYP17A1 (c,d) and CYP19A1 (e,f) was evaluated in hGLC after 8 h (a,c,e) and 24 h (b,d,f) of gonadotropins treatment in the presence or in the absence of BaP. Data are represented over the housekeeping gene RPS7, and as mean ± SEM. *, Significantly different vs “no hormone”; (Kruskal Wallis test and Dunn’s post-hoc test, *p < 0.05; n=6).

**Supplementary Figure 7. BaP effect on 8 h-steroids production in mLTC1 and hGLC.** The effect of BaP on progesterone (a) and testosterone (b) levels in mLTC1 cells (n=8) and progesterone (c) and estradiol (d) levels in hGLC (n=10) was evaluated after 8 h treatment of LH and hCG. Data are represented as mean ± SEM. *, Significantly different vs “no hormone”; (Kruskal Wallis and Dunn’s post-hoc test, *p < 0.05).
