# Supplementary figures and images for: Benzo[a]pyrene disrupts LH/hCG-dependent mouse Leydig cell steroidogenesis through receptor/Gαs protein targeting
Source: Sci Rep. 2024 Jan 8;14:844. doi: 10.1038/s41598-024-51516-7 (PMC10774265; doi:10.1038/s41598-024-51516-7)

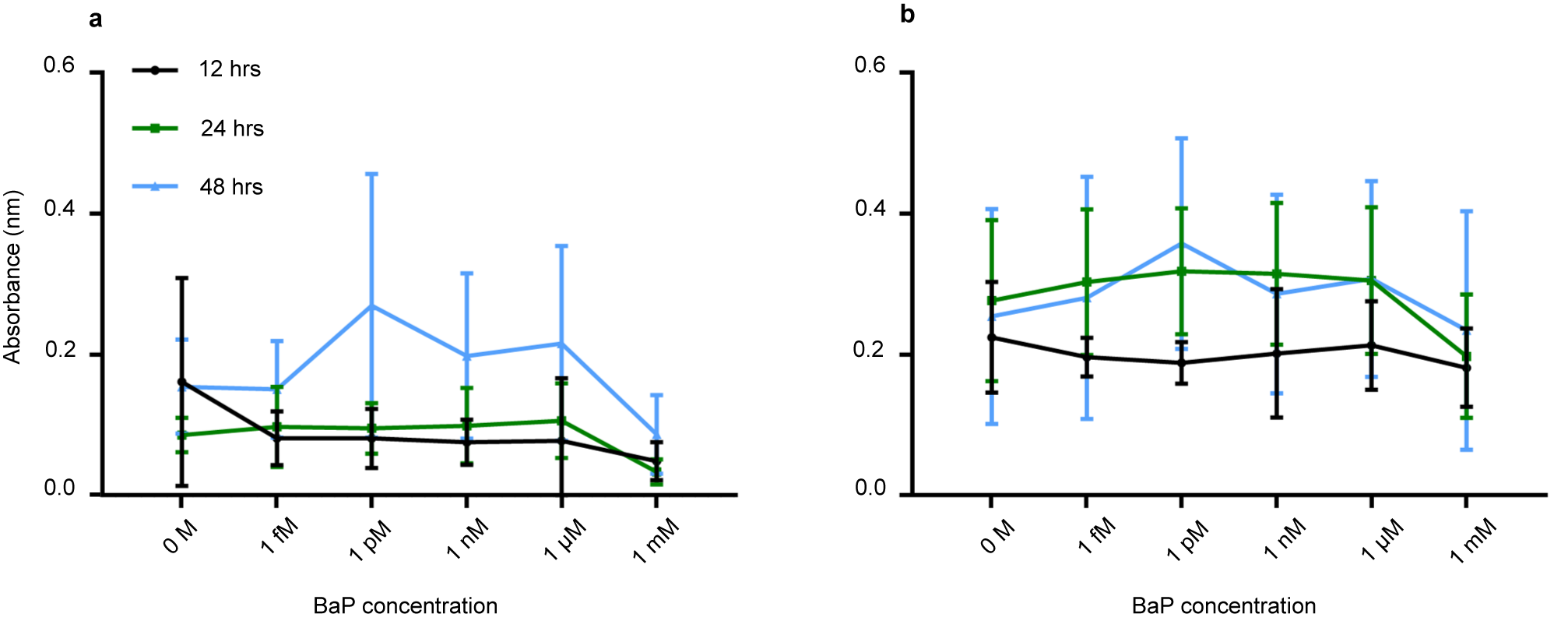

Supplement: Supplementary file 2 — Supplementary Figure 1. [file 41598_2024_51516_MOESM2_ESM.tif]

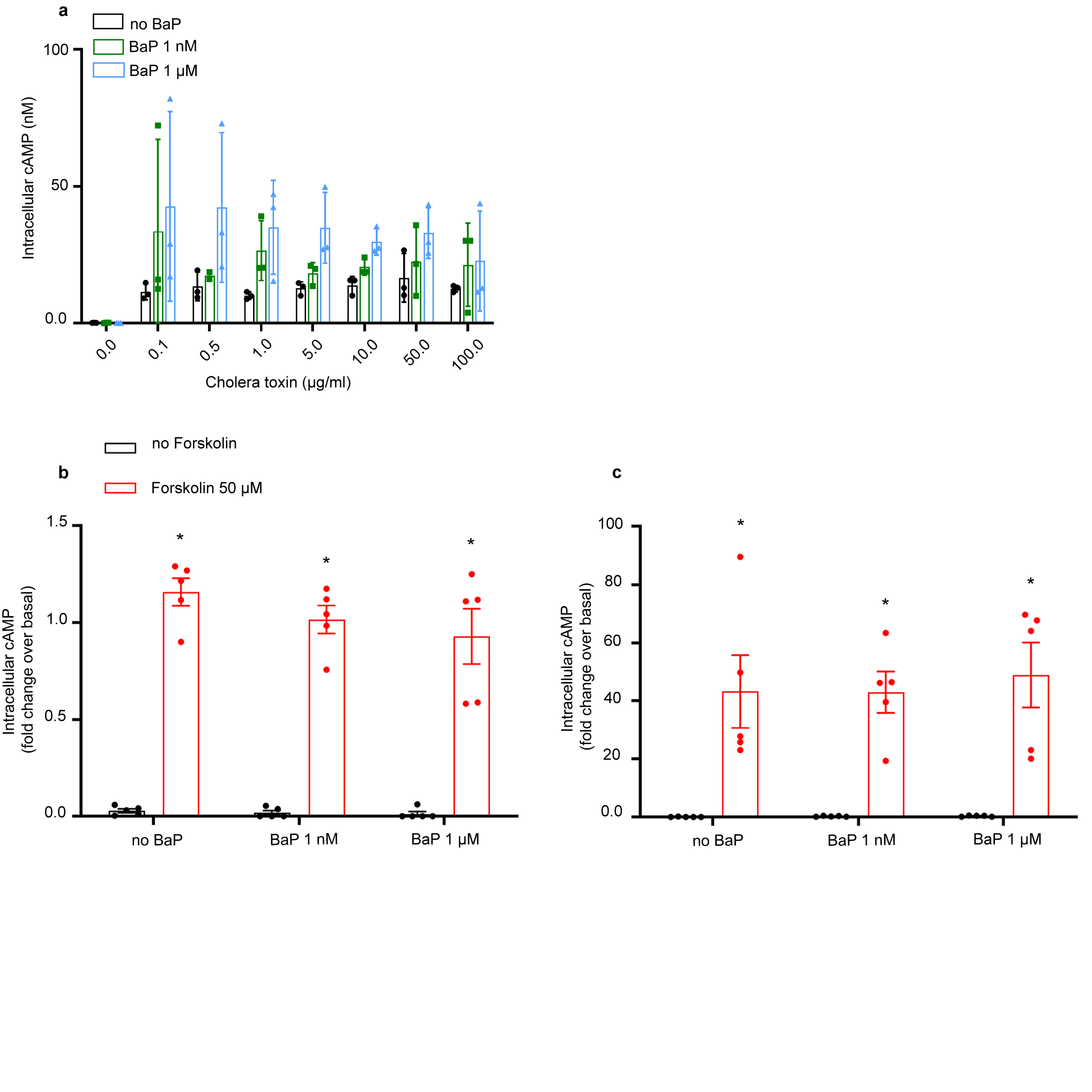

Supplement: Supplementary file 3 — Supplementary Figure 2. [file 41598_2024_51516_MOESM3_ESM.tif]

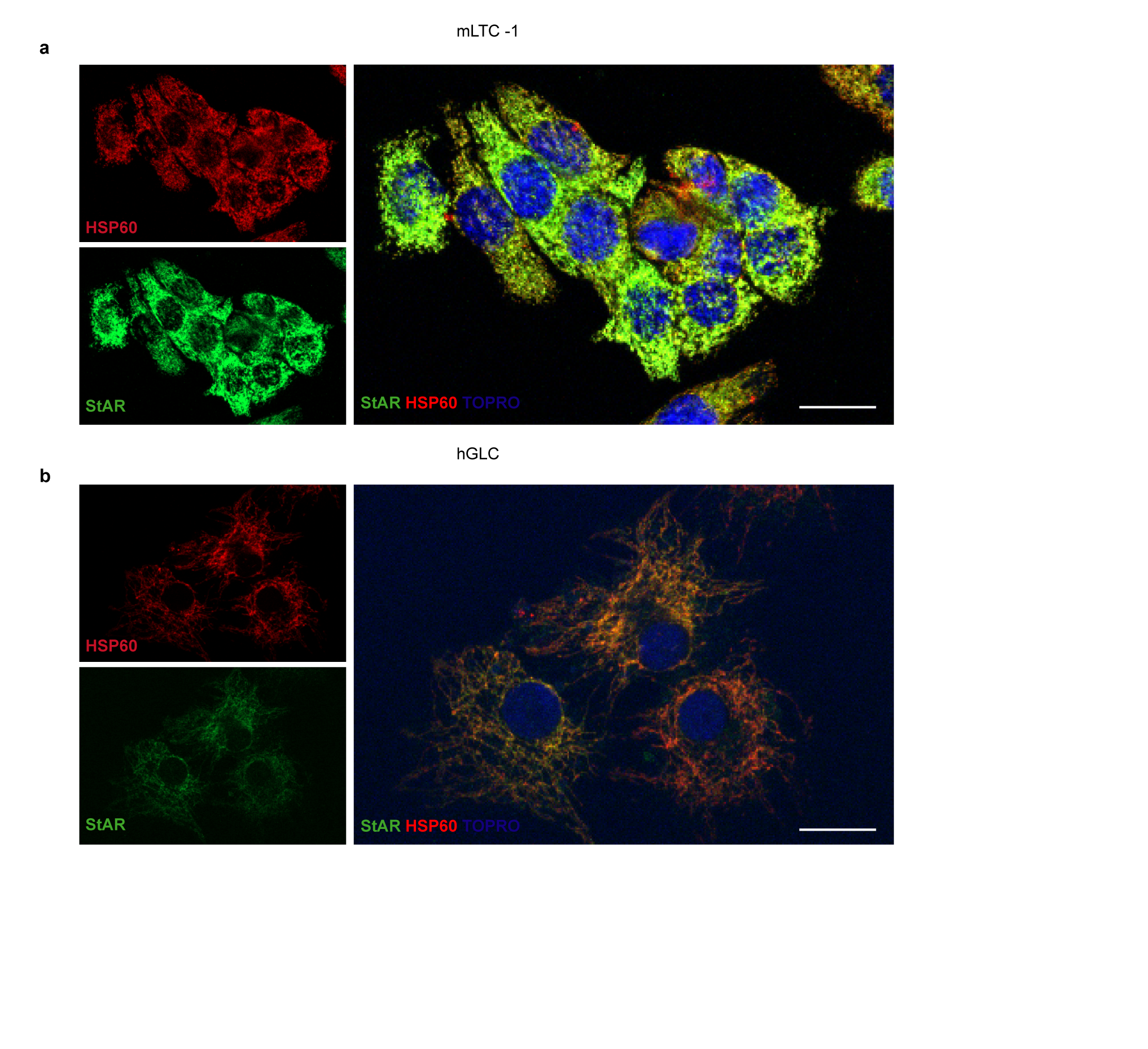

Supplement: Supplementary file 4 — Supplementary Figure 3. [file 41598_2024_51516_MOESM4_ESM.tif]

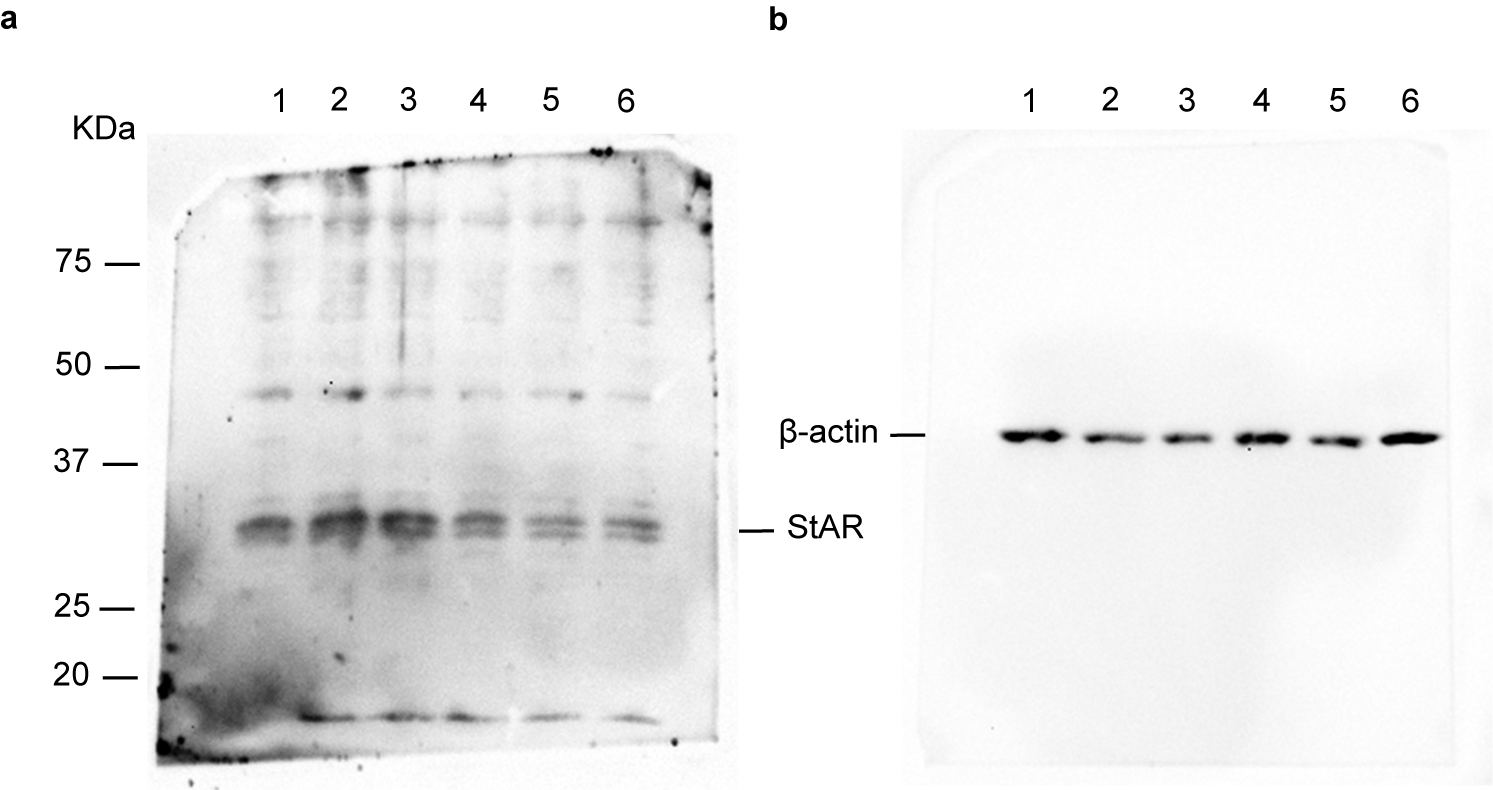

Supplement: Supplementary file 5 — Supplementary Figure 4. [file 41598_2024_51516_MOESM5_ESM.tif]

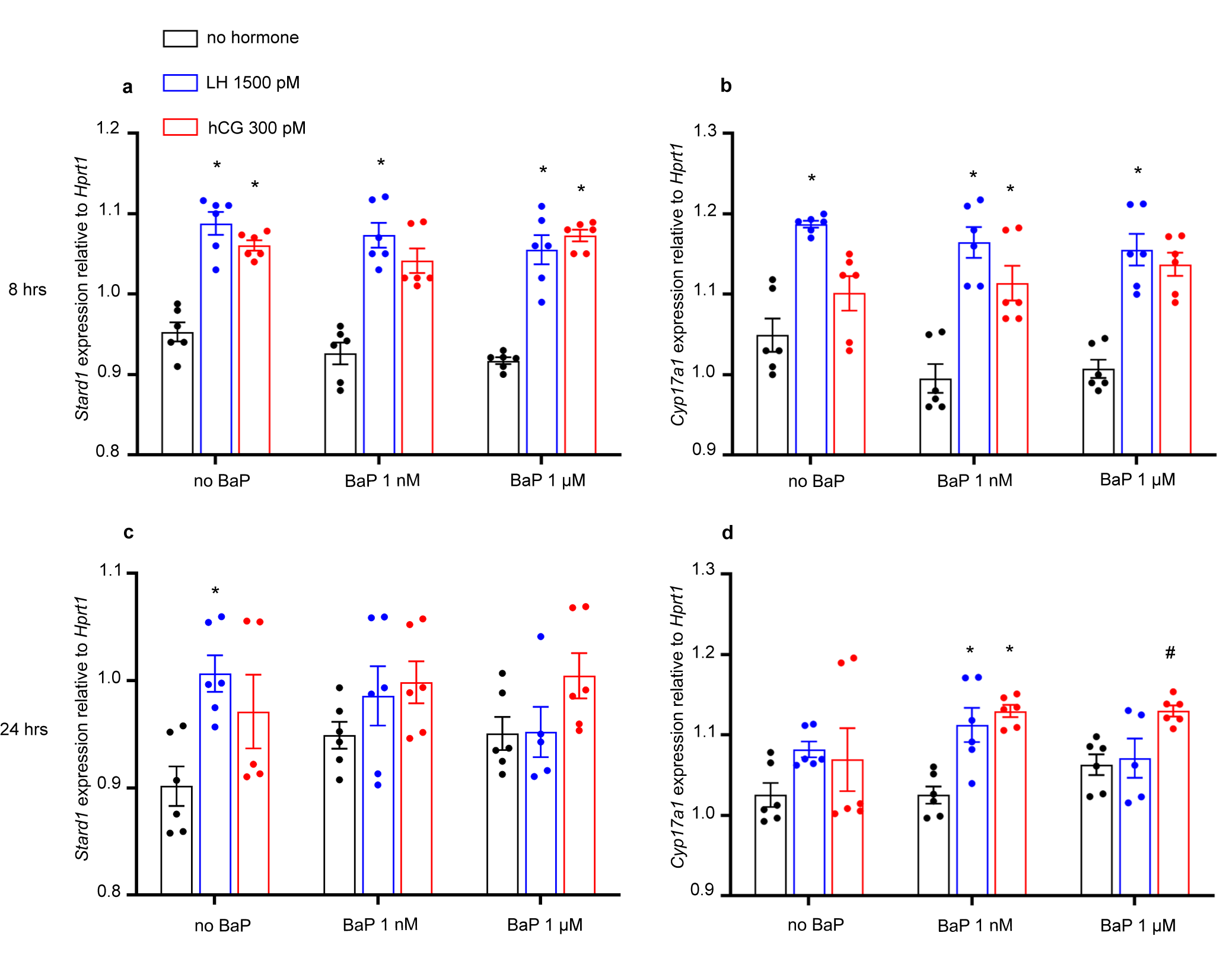

Supplement: Supplementary file 6 — Supplementary Figure 5. [file 41598_2024_51516_MOESM6_ESM.tif]

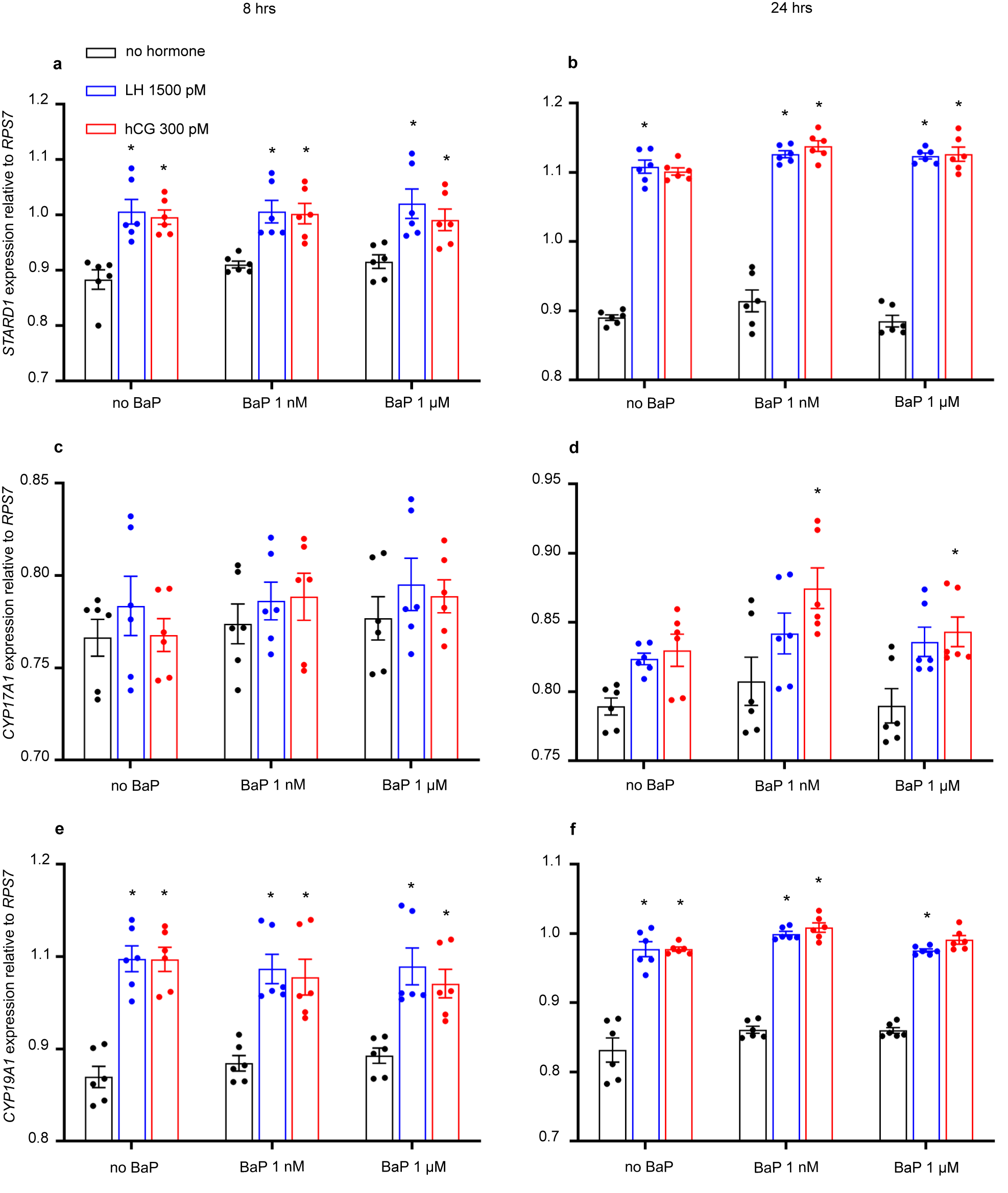

Supplement: Supplementary file 7 — Supplementary Figure 6. [file 41598_2024_51516_MOESM7_ESM.tif]

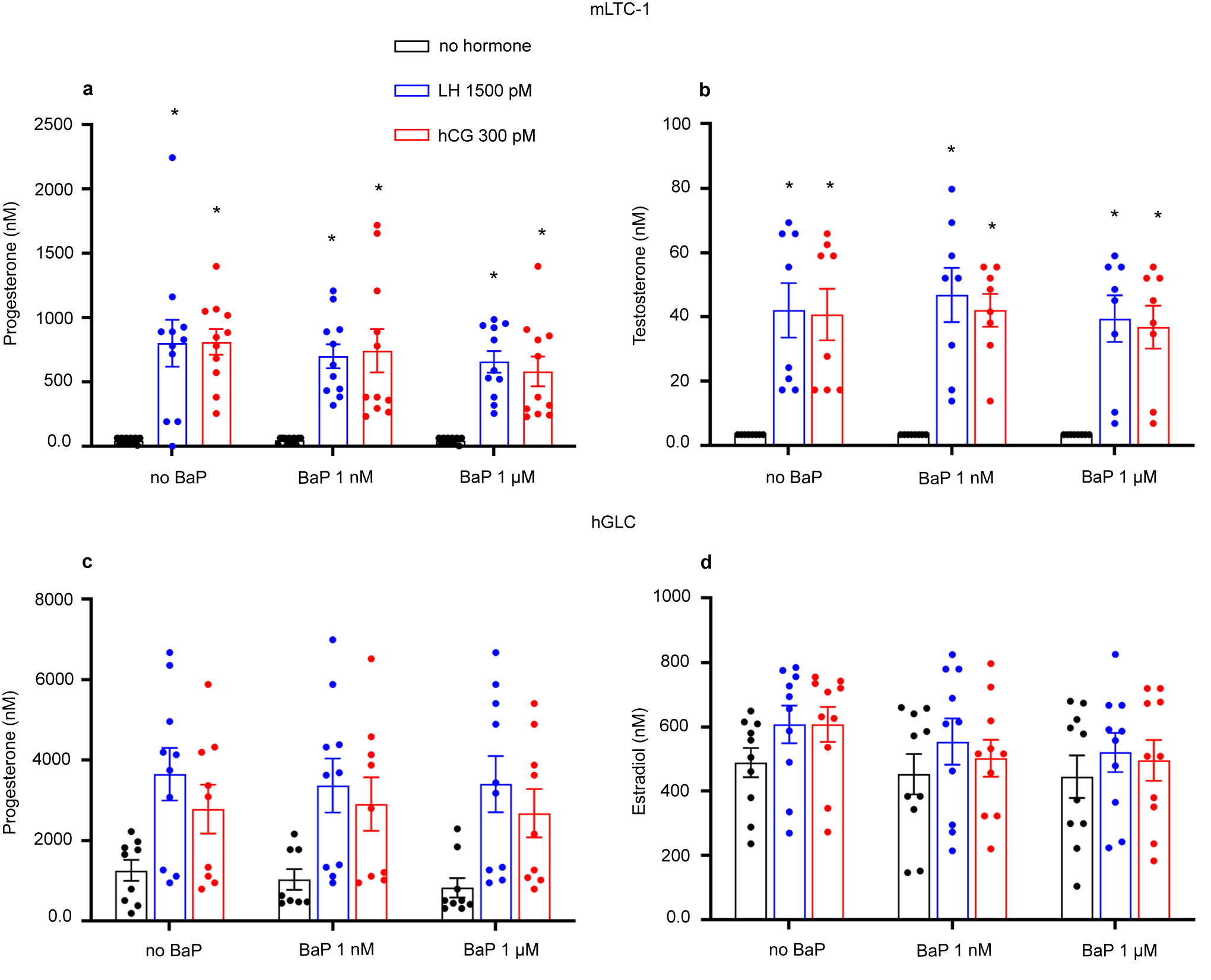

Supplement: Supplementary file 8 — Supplementary Figure 7. [file 41598_2024_51516_MOESM8_ESM.tif]
