# Supplementary material for: Benzo[a]pyrene disrupts LH/hCG-dependent mouse Leydig cell steroidogenesis through receptor/Gαs protein targeting
Source: Sci Rep. 2024 Jan 8;14:844. doi: 10.1038/s41598-024-51516-7 (PMC10774265; doi:10.1038/s41598-024-51516-7)
Supplement: Supplementary file 9 — Supplementary Information. [file 41598_2024_51516_MOESM9_ESM.pdf]

Supplemental Information: Full Original Blot Images

The original images of the western blotting analysis reported in Figure 2a and 2b of the manuscript are shown below. All the membranes were cut before labelling with antibodies, while they were not further modified after the assessment with the antibody.

Original blots for membranes presented in Figure 2a:

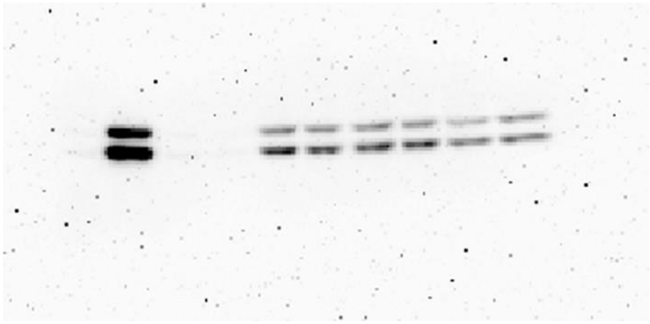

Analysis of pERK1/2 in mLTC1

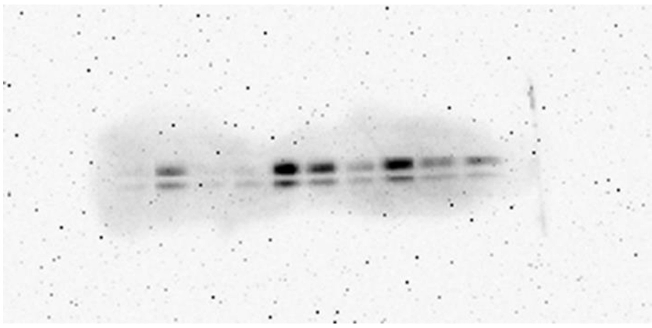

Analysis of pCREB in mLTC1

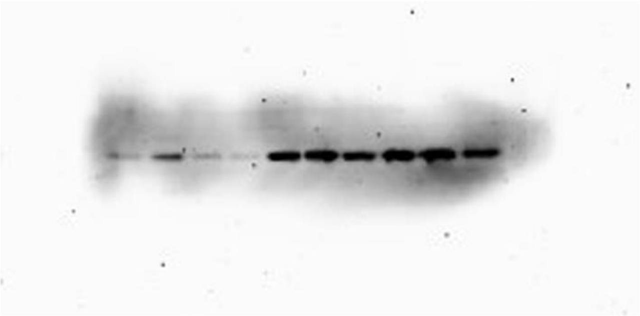

Analysis of p38 MAPK in mLTC1

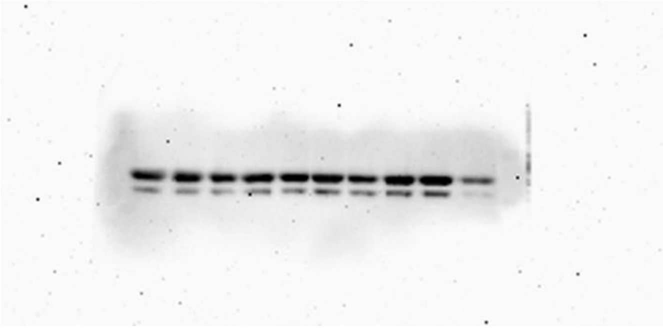

Analysis of total ERK in mLTC1

Original blots for membranes presented in Figure 2b:

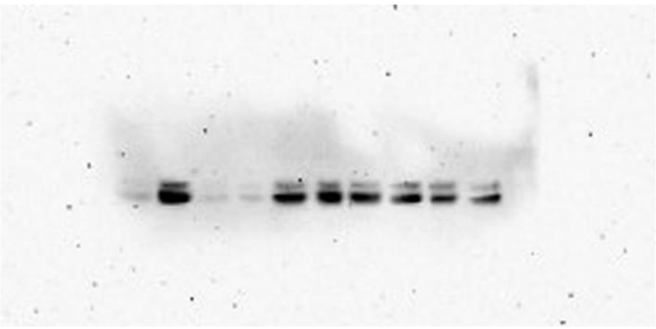

Analysis of pERK1/2 in hGLC

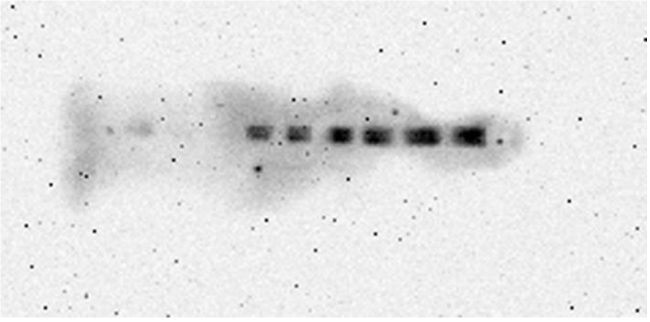

Analysis of pCREB in hGLC

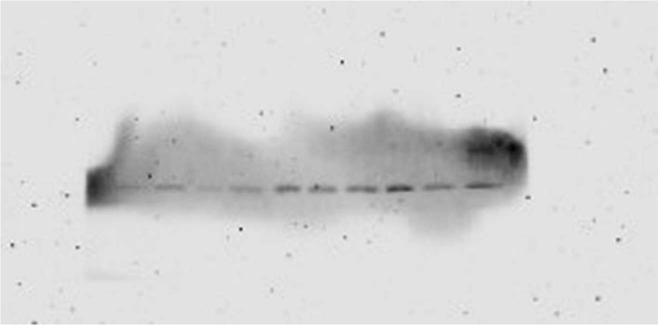

Analysis of p38 MAPK in hGLC

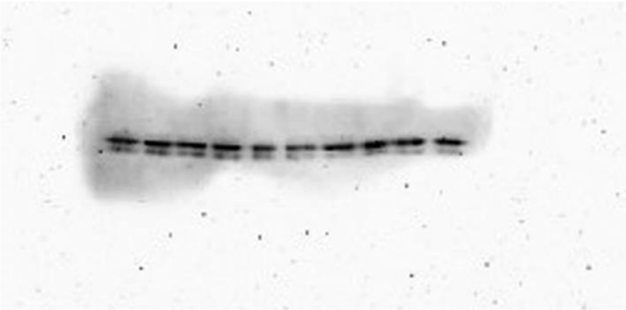

Analysis of tot ERK in hGLC
